# Factors affecting dental diseases presenting at the University of Ghana Hospital

**DOI:** 10.1186/s40064-016-3391-y

**Published:** 2016-10-04

**Authors:** Joseph Nimako-Boateng, Michael Owusu-Antwi, Priscilla Nortey

**Affiliations:** 1 University of Ghana Hospital, Legon, Ghana; 2 University of Ghana School of Public Health, Legon, Ghana

**Keywords:** Dental diseases, Acute conditions, Chronic condition

## Abstract

Dental diseases are common in man and range from a toothache to cancers of the head and neck. Dental conditions can affect our capacity to function effectively in areas such as smiling, chewing and speaking. The objective of this study was to describe the main types of dental conditions presenting at the University Hospital between January 2006 and December 2011 and to determine factors associated with the top five diagnosed conditions as well as the acute and chronic conditions. A retrospective review of all 5012 clinical records of dental patients visiting the dental unit within the period stated was carried out. A total of 4196 records which passed the inclusion/exclusion criteria were analysed. Most of the patients who presented were adults. The top 5 dental conditions were apical periodontitis (50.4 %), reversible pulpitis (23.3 %), Gingivitis (11.4 %), Periodontitis (6.2 %) and Halitosis/bad breadth (8.8 %). The top 5 conditions constituted over 75 % of the cases seen. About 84 % of the presentation was acute whilst 16 % was chronic. For the entire dataset and also the top five conditions, approximately 53.0 % were males and 47.0 %. Males outnumbered females on all occasions except for halitosis and most patients presented within 1 month of experiencing symptoms of dental disease. Some significant association was found between the presence of multiple chronic conditions and reversible/irreversible pulpitis. The main dental conditions presenting at the University Hospital during the stated period (i.e. between January 2006 and December 2011) were: apical periodontitis (50.4 %), reversible pulpitis (23.3 %), gingivitis (11.4 %), periodontitis (6.2 %) and halitosis/bad breadth (8.8 %).

## Background

The most common diseases in man include dental diseases such as gingivitis and caries. Different parts of the mouth such as the teeth, gums or other tissues may be affected by dental disease. Apart from a toothache, which is a commonly experienced dental disease, others may manifest as inability to speak, smile or even chew with a varying degrees of severity (Sanu et al. 2010). Dental disease affects people of all age groups and all races. It affects both adults and children, deciduous and permanent dentition. Although when dental disease is left undiagnosed and untreated could lead to life threatening conditions there is a preponderance of dental diseases among the poor and disadvantaged groups worldwide (Upadhyaya and Humagain 2009). Dental caries and periodontal disease are the two most common dental diseases of importance to public health worldwide although there are several others, which have both clinical, and public health importance (Varenne et al. 2005). The distribution of dental diseases varies internationally, inter regionally and inter-continentally (Petersen 2004). For example within Europe and the Americas, dental diseases are more common in minority groups and African Americans. With dental caries affecting 60–90 % of schoolchildren and many adults, industrialized countries retain dental caries as a major health problem. Although incidence of caries appears to be lower in most African countries this is predicted to rise due to increasing consumption of sugars and decreasing exposure to fluorides according to Petersen (2004) and Varenne et al. (2005).

### Overview of dental diseases

The two most common dental conditions of importance to public health are outlined in this section. ‘Diet and dirt’ have been named as the culprits for most dental conditions worldwide.

### Dental caries

Dental caries refers to enamel, dentine or cementum destruction bacterial acid produced in dental plaque leading to a cavity in the tooth crown or root (Selwitz et al. 2007; Marsh and Martin 1999). Usually, dental caries progresses slowly as a chronic disease although its rate of progress varies. There are phases of quiescence during which the caries gets arrested or re-mineralized. The result is that a decayed cavity may not change for years.

Though less common, rampant caries sometimes occurs which refers to a progressively rapid destruction of tooth tissue during more active phases of the carious lesion. Dental caries in man follows a characteristic pattern. Molars are more frequently affected; anterior teeth are least affected with premolars somewhere in between (Kidd and Fejerskov 2003).

Commonly carious lesions are coronal in nature and affect the pits and fissures of premolars and molars. Proximal crown surfaces of posterior and anterior teeth typically experience fewer caries. Carious lesions in the lingual, labial and buccal surfaces of teeth are fairly uncommon. Coronal caries are more common amongst children and younger adults whilst root caries is commonly found among older adults who retain their teeth for longer. Girls experience more caries than boys probably because girls develop teeth faster than boys (Selwitz et al. 2007; Marsh and Martin 1999).

Sugars can be classified as intrinsic or extrinsic. Intrinsic sugars are found within the body of fruits such as oranges, mangoes, watermelon whilst extrinsic sugars referred to refined sugars which are added to food, snacks etc. Extrinsic sugars found in milk and intrinsic sugars are generally known to be non-cariogenic (Kidd and Fejerskov 2003).

One predominantly outstanding factor in the epidemiology of caries is non-milk extrinsic sugars and the evidence for this lies in the multiplicity of studies, which have confirmed this over several decades. This is demonstrated by the fact that sugar rationing in Japan, Norway and the Island of Jersey during World War II was accompanied by a decline in the rate of caries (Marsh and Martin 1999).

### Periodontal disease

Periodontal disease typically affects structures which support the teeth. It ranges from a mild gingivitis to a more severe pattern of bone loss. Bone is lost in two ways—vertically and horizontally (Page and Schroeder 1976; Offenbacher 1996). When bone is lost horizontally, several teeth are involved as compared to vertically where one or two teeth are involved. Furthermore when bone is lost horizontally, there is loss in height of bone around all the four walls of the teeth whilst there are intra-bony defects surrounded by high walls of unaffected bone as part of vertical bone loss (Page and Schroeder 1976).

The bone loss usually affects the first and second molars in a symmetrical fashion and is clearly associated with age although the causes of periodontitis are multifactorial. The condition is more common in adults than it is in children (Offenbacher 1996).

### Prevention of dental diseases

Prevention of dental diseases has followed different approaches such as community based interventions, individual measures, the common risk factor approach as well as targeting at risk groups. Community water fluoridation, salt fluoridation, milk fluoridation as well as school based placement of fissure sealants are some of the broad community measures that have been trialed in preventing dental diseases such as dental caries (Marinho et al. 2003; Batchelor and Sheiham 2002). Tooth brushing, flossing and regular dental visits are some preventative measures that can be taken by an individual to avoid dental diseases. Smoking cessation support is a common risk factor approach in preventing oral cancers, halitosis, periodontal disease, lung cancers, hypertension and several other ailments which are caused by tobacco smoking. Since dental disease is unevenly distributed within populations, strategic targeted interventions could be planned for children in deprived communities where dental disease is high (Marinho et al. 2003; Batchelor and Sheiham 2002).

### Diagnosing dental diseases

Dental diseases are diagnosed by collating good clinical history and the carrying out a clinical examination (Ismail 2004; Pitts 1997). This may be supported in most cases by radiographic examinations of the teeth and the jaws for underlying pathology which may not be readily obvious (Ismail 2004).

### The global picture of dental diseases

The decline of dental caries in children in economies such as North America, Western Europe, Canada, New Zealand, Japan and Australia, has been well documented and here, caries prevalence and incidence had dropped dramatically amongst 12 year olds. However this trend is reversed in Africa and other developing economies (Whelton 2004).

### Dental diseases Europe and America

Dental diseases in Europe and America have been better researched and documented compared with other regions of the world. The patterns and trends of dental and oral diseases are changing because the middle-aged and older adults are keeping their teeth for longer (Upadhyaya and Humagain 2009). Since fewer numbers of adults are edentulous, more adults are requiring and demanding dental care. Although the trends have shown a decline in the severity of dental caries in most children, the need for dental care amongst middle-aged and older adults has increased. It must be noted that dental caries has not declined for lower-income, certain minority and immigrant groups of children (Douglass and Sheets 2000; Kim et al. 2012; Okunseri et al. 2012). Poverty, minority status, appears to be one of the strongest predictors of dental caries in Europe and the United States of America both in children and adults. Oral Health inequalities abound as a myriad of studies show that access is also not readily available for the poor and deprived of these developed economies (Douglass and Sheets 2000; Schimmel et al. 2008; Luzzi and Spencer 2008).

### Dental diseases in Africa and Asia

Most patients with dental problems in Africa and Asia present late with acute pain especially amongst the poor and deprived of these populations and with these sub-groups opting more for extractions which appear to be the norm. Although aesthetics is not a high priority for the poor patient in this setting and in selecting prosthetic replacement Kenndy’s class III denture is the choice for cost and aesthetic reasons (Ajayi et al. 2012; Sanu et al. 2010; Varenne et al. 2005; Ehikhamenor et al. 2010; Upadhyaya and Humagain 2009). Poor access and low utilisation of dental services due to anticipation of high charges, painful treatment, long waiting times among others is the hallmark of most government dental facilities within the African context (Bamise et al. 2008; Åstrøm and Masalu 2001). Although global cancer statistics indicates that India has one of the highest incidence rates for oral cancer worldwide, overall, the awareness of oral cancer and its associated risk is relatively low (Agrawal et al. 2012).

### Dental diseases in the Ghanaian context

One of the medium term objectives of oral health in Ghana is improving and strengthening data collection systems and research in oral health according to the Oral Health Unit of the Ghana Health Service report in 2011. This is because there is very little information available to assist decision makers. Information that is produced is often too late to assist/input into decision making, or may be of dubious accuracy. The need for an effective and efficient oral health information system is vital for planning, good decision-making, ongoing monitoring and evaluation and to improve coverage and quality of care.

According to the 2007–2011 draft oral health policy, oral health care in Ghana has not seen much improvement over the years. The two main oral health diseases of public health importance are caries and periodontal disease (1998 Oral Health Unit Report). In 2006, dental caries accounted for 80 % of dental extractions performed in Public Dental Clinics whilst periodontal disease accounted for 13 %.

Calculus still remains the commonest presentation of gum disease. In 2003, 73 % at age 12, 56 % of adults aged 35–44 and 74 % of adults aged 55+ have calculus.

Studies reviewing the pattern of all oral diseases presented at any specified dental clinics are so few and virtually non-existent. Most of the research done involves the selection of one condition or a particular age group such as children and adolescents. This study provides a composite picture of all the diseases presenting at the Dental Unit of the University Hospital and enables us to describe the top five conditions, categorizing them into acute or chronic and assess any associations with the past medical history.

## Methods

### Study site

The study was conducted at the Dental Unit, of the University Hospital, Legon. The University of Ghana Hospital was built to cater for the health needs of students, staff and their dependants. Over time the hospital has assumed the functions of a district hospital and has a wide catchment area. This quasi-government institution has medical, surgical, maternity, paediatric, casualty and emergency, dental, operating theatre and public health units and a bed capacity of 121. To meet the growing demand for specialist services, specialist consultancy services such as paediatrics, general surgery, obstetrics and gynaecology, orthopaedic surgery were introduced in 1996/97 and the dental unit started as part of the specialist consultancy services that same year. A full dental wing was constructed after which regular dental services was started in 2005. The University Hospital Dental Unit is a general dental practice providing first primary dental care, and patients presenting a range of conditions.

The university classifies staff and students into the following groups; junior members (students), junior staff, senior staff, senior members and private patients. “Staff” means a person other than senior members in the employment of the University. “Junior Staff” are members of staff below the rank of Administrative Assistant or its equivalent. “Senior Staff” means member of staff not below the rank of Administrative Assistant or its equivalent.

A “Junior Member” means a person in status pupillary enrolled for the time being in the University, whether in a campus based or distance education programme. “Senior Member” means the academic, administrative, professional employees and members of convocation or persons who would become members if they were not of <2 years standing from their first degrees or equivalent qualifications. Broadly speaking, “private patients” are those who are not staff or members or dependants of the staff and senior members and therefore are not associated with the university.

### Study design

A retrospective review of clinical records of dental patients who visited the dental unit of the University Hospital between January 2006 and December 2011 was conducted.

### Study population

All the records of patients seen from January 2006 till December 2011 were obtained. The population comprised consecutive records of all patients seen over this period.

### Data collection

Secondary data made of patients records was retrieved by trained data collectors. The training was done by the principal investigator using a sample patient record. All the parameters listed in the folder were explained to them to facilitate the understanding of the information to be captured. Data collectors were graduates who were awaiting their national service.

### Inclusion criteria

In order to get an all-inclusive population, all the records of patients seen within the period 2006–2011 were included in the study. Therefore a total of 5012 paper based consecutive records was retrieved and entered into the data collection sheets.

### Exclusion criteria

Any record without a diagnosis, age and sex was excluded from the analyses. This was done because the dependent variable is the diagnoses and some demographic inputs will be required in its analyses. In total, 4196 records made it to the analyses stage because they met the inclusion and exclusion criteria whilst 816 paper based records were omitted from the study.

### Description of variables

The dependent variable was diagnoses such as dentoalveolar abcess, irreversible pulpitis, enamel hypoplasia and reversible pulpitis. The full list of the dependent variables as illustrated in Table 3 are:

1. Apical periodontitis and or irreversible pulpitis;
2. Reversible pulpitis;
3. Gingivitis;
4. Periodontitis;
5. Halitosis/bad breath;
6. Impacted tooth/pericoronitis;
7. Dentoalveolar abcess;
8. Maxillary sinusitis;
9. Mucosal ulceration/lesion;
10. Fractured tooth;
11. No abnormality found;
12. Others e.g. locked jaw, ameloblastomas.

Whilst an acute condition will have symptoms appearing and changing rapidly, a chronic condition will develop and worsen over an extended period of time. Therefore duration and presentation of the symptoms aided in categorizing the condition as acute such as (irreversible pulpitis) and chronic (reversible pulpitis). It is important to note that depending on the duration of the symptom, a condition such as a fractured incisor may be categorized as acute or chronic. Where the condition is better classified as acute or chronic, it was added to the acute group since it was the acute exacerbation that precipitated the visit.

The independent variables are as follows; age (e.g. 0–10, 11–20), sex, associated professional category (e.g. student, junior staff, and private patients). Other independent variables include chief complaint characteristic (e.g. toothache, bleeding gums and bad breadth), duration of chief complaint (e.g. <1 week, 1 week–1 month), past medical history (e.g. diabetic, hypertensive), past dental history (e.g. simple filling, Root canal treatment). The rest are the conditions of the oral mucosa (e.g. gingivitis, ulcerated), condition of the teeth (e.g. carious, fractured), investigations (e.g. periapicals, fasting blood sugar) and the first item on the treatment plan (e.g. scaling and polishing, simple filling).

## Empirical analysis and discussion

This section describes the output obtained by analysing the data obtained from the university hospital. The total sample size is 4196 out of 5012 records which contain demographic information of patients, the various diagnoses of patients, chief complaints, investigations and other relevant information. The analysis is in two sections; that is, descriptive statistics and some tests of relationships between certain variables (Chi square test of independence).

The decline in the numbers seen from 2006 till 2009/2010 for most of the categories may probably reflect the staffing and enrolment status of the university as a whole.

### Characteristics of sample

A sample of 4196 was selected based on already predefined inclusion and exclusion criteria from all patients’ records available from 2006 to 2011. Table 1 shows that out of the 4196, 52.9 % were males whilst 47.1 % were females. This distribution could be due to the male to female ratio within the University of Ghana although this could also be attributable to gender specific health attitudes reported by Al-Omari and Hamasha (2005) which will predispose males to more oral health problems.

**Table 1:** Patient categories by year

Year	Private patient	Senior staff	Senior member	Junior staff	Student	Total
2006	358	83	85	73	223	822
2007	233	65	56	51	224	629
2008	182	71	36	57	131	477
2009	291	51	50	61	266	719
2010	529	51	48	55	274	957
2011	317	41	27	48	105	592
Total	1964	362	302	345	1223	4196

The top 5 conditions constitute over 75 % of the cases seen and have similar male to female distribution as the entire data set. About 84 % showed acute presentations whilst 16 % showed chronic presentations. The predominance of acute presentations shows the fact that patients usually present when they are in pain or significant discomfort.

The male to female proportions in both the acute and chronic group are almost evenly distributed.

The young adults and older adults together constitute approximately 70 % of the cases seen for the entire data set and the top 5 acute and chronic conditions as well. This is consistent with the population profile of the University of Ghana. The age group which least patronized the clinic was the elderly group which averagely contributes about 3–5 % of the various sub-groups already described. This finding is consistent with Bhadbhade (2015), who noted that elderly persons are less likely to visit the dental clinic and are less knowledgeable about dental diseases.

As illustrated in Table 2, the private patients constituted about 45 % of all patients seen for the entire data and the top 5. Students who constitute about 30 % for the two groups described closely follow this and the acute/chronic presentation followed a similar pattern. This also is an indication of the fact that the residents in its catchment area actively patronize the Dental Unit of the University Hospital. The high numbers of private patients acts as an indicator of the need in the catchment area for the hospital. The student population in the University is about forty thousand and this explains why we have the next largest patient category being students.

**Table 2:** Demographic characteristics of patients

Characteristics	All presenting dental diseases (%)	Top five dental diseases N (%)	Acute conditions (%)	Chronic conditions (%)
Sex				
Male	1978 (47.1)	1706 (47.2)	1054 (40.1)	193 (7.3)
Female	2218 (52.9)	1908 (52.8)	1139 (43.4)	242 (9.2)
Total	4196 (100.0)	3614 (100.0)	2194 (83.5)	435 (16.5)
Age at first visit				
Children (<11)	342 (8.2)	291 (8.0)	167 (7.6)	23 (5.3)
Adolescents (11–20)	707 (16.8)	629 (17.4)	377 (17.2)	70 (16.1)
Young adults (21–40)	2145 (51.1)	1855 (51.3)	1148 (52.3)	262(60.2)
Older adults (41–60)	763 (18.2)	646 (17.9)	386 (17.6)	65 (14.9)
Elderly (>60)	239 (5.7)	194 (5.4)	116 (5.3)	15 (3.4)
Patient category				
Private patients	1964 (46.81)	1613 (44.6)	1141 (43.40	149 (5.67)
Senior staff	362 (8.63)	275 (7.6)	134 (5.10)	29 (1.10)
Senior member	302 (7.20)	299 (8.3)	155 (5.90)	32 (1.22)
Junior staff	345 (8.21)	322 (8.9)	217 (8.25)	26 (0.99)
Students	1223 (29.15)	1106 (30.6)	547 (20.81)	199 (7.57)

The top five dental conditions seen here are a reflection of patients’ attitude to dentistry as a whole. Most people attend the clinic when they are in pain and are unable to chew. At that stage of apical periodontitis, patients are presented with the unpleasant options of either carrying out a root canal or an extraction.

### Summary of all diagnoses

Table 3 presents the diagnoses obtained from our entire data set from which the top 5 diagnoses were extracted.

**Table 3:** All diagnoses

Diagnoses	Frequency	%
Apical periodontitis	1822	43.4
Reversible pulpitis	842	20.1
Gingivitis	411	9.8
Periodontitis	223	5.3
Halitosis/bad breath	317	7.6
Impacted tooth/pericoronitis	56	1.3
Dentoalveolar abcess	65	1.5
Maxillary sinusitis	44	1.0
Mucosal ulceration/lesion	50	1.2
Fractured tooth	124	3.0
No abnormality found	126	3.0
Others e.g. locked jaw, ameloblastomas	116	2.8
Total	4196	100.0

It is important to note that in cases where a patient presented with multiple diagnoses, the diagnosis most consistent with the key presenting complaint during the first visit was selected. In the very few cases where this was not possible, a diagnosis was randomly chosen out of the lot.

### The top 5 dental conditions

Table 3 above clearly shows the top 5 dental conditions. It shows that apical periodontitis is the leading condition with 43.4 % followed by reversible pulpitis with 20.1 %. This is consistent with similar findings of Whyman et al. (1996) in the Auckland Hospital Clinic where gingivitis, halitosis and periodontitis followed with 9.8, 7.6 and 5.3 % respectively. Most of the patients who presented with each of the constituent top 5 conditions as well as acute and chronic presentation were young or older adults whilst elderly patients were the least to present. Males outnumbered females on all occasions except for halitosis where females outnumbered the males. Most patients presented within 1 month of experiencing symptoms of dental disease (Table 4).

**Table 4:** Top 5 acute and chronic conditions and past medical and dental histories

Characteristics	Apical periodontitis	Reversible pulpitis	Gingivitis	Periodontitis	Halitosis	Acute	Chronic
Past medical history							
Hypertension	167 (9.2)	76 (9.0)	27 (6.6)	19 (8.5)	23 (7.3)	193 (7.34)	27 (1.03)
Diabetic	134 (7.4)	50 (5.9)	21 (5.1)	15 (6.7)	25 (7.9)	141 (5.36)	27 (1.03)
Others	79 (4.3)	43 (5.1)	20 (4.9)	7 (3.1)	17 (5.4)	107 (4.07)	24 (0.91)
Nil of note	1442 (79.1)	673 (79.9)	343 (83.5)	182 (81.6)	252 (79.5)	1753 (66.68)	357 (13.58)
Total	1822 (100)	842 (100)	411 (100)	223 (100)	317 (100)	2194 (83.5)	435 (16.5)
Past dental history							
No PDH	960 (52.7)	497 (59.0)	198 (48.2)	143 (64.1)	179 (56.5)	1158 (44.05)	243 (9.24)
History of previous extraction	354 (19.4)	141 (16.7)	82 (20.0)	43 (19.3)	62 (19.6)	467 (17.76)	75 (2.85)
History of routine preventive care	221 (12.1)	83 (9.9)	69 (16.8)	26 (11.7)	33 (10.4)	261 (9.93)	58 (2.21)
History of all other treatments	287 (15.8)	121 (14.4)	62 (15.1)	11 (4.9)	43 (13.6)	308 (11.72)	59 (2.24)
Total	1822 (100.0)	842 (100.0)	411 (100.0)	223 (100.0)	317 (100)	2194 (83.5)	435 (16.5)
Multiple chronic conditions							
Yes	40 (2.20)	18 (2.14)	7 (1.70)	6 (2.69)	2 (0.63)	73 (3.33)	6 (1.38)
No	1782 (97.8)	824 (97.86)	404 (98.3)	218 (97.31)	315 (99.37)	2121 (96.67)	429 (98.62)

As illustrated in Table 4, most of the patients presenting with the various dental conditions had no significant medical or dental history or multiple chronic condition. However hypertension and diabetes were the commonest medical history elicited in all the categories. The commonest dental history was extraction consistent with the findings of Whyman et al. (1996) and the least common dental history was routine preventive care except in gingivitis and periodontitis which is unlike previous findings of Bhadbhade (2015) where all other treatments were less than that of preventive care (Table 5).

**Table 5:** Chi square test of relationship between condition (acute or chronic) and demographics

Diagnosis	Characteristics									
	Age groups		Duration of symptom		PMH		PDH		Multiple chronic	
	Chi square	p value	Chi square	p value	Chi square	p value	Chi square	p value	Chi square	p value
Apical periodontitis	18.113	0.001	13.265	0.001	1.888	0.389	6.168	0.46	19.462	0.000
Enamel caries	6.116	0.191	10.839	0.004	0.301	0.860	8.432	0.015	9.418	0.002
Gingivitis	10.588	0.032	3.641	0.162	3.767	0.152	9.054	0.011	0.147	0.701
Periodontitis	2.723	0.605	0.088	0.957	0.554	0.758	12.617	0.002	1.264	0.261
Halitosis	0.035	1.000	9.985	0.010	1.853	0.396	1.091	0.579	3.332	0.068
Severity of condition	11.149	0.025	21.753	0.000	3.179	0.204	3.655	0.161	13.615	0.000

The factors of age, duration of symptoms as well as the presence of multiple chronic conditions is related to the diagnoses arrived at during the dental visit as shown in Table 5.

The following conclusions are drawn from the test of relationship between the top five diagnoses, the severity of condition and some variables. p values <0.05 show significant relationships between the two variables. With respect to age, the p value of the Chi square test above shows that the age group of patients is related to the condition of apical periodontitis and gingivitis but not enamel caries, periodontitis and halitosis. It also shows that their various age group is related to the severity of their condition. The p value of the Chi square test above shows that the duration of symptom of patients is related to the condition of enamel caries, halitosis and the severity of the presenting condition but not apical periodontitis, gingivitis, enamel caries and periodontitis.

Although the tests above shows some significant associations between past dental history on one hand and gingivitis, periodontitis and reversible pulpitis, on the other hand no relationship was found with halitosis, apical periodontitis and severity of presentation. Past medical history did not indicate any relationship with any of the parameters described. The p value of the Chi square test above shows that the multiple chronic conditions of patients is related to apical periodontitis, enamel caries and the severity of presenting complaint but it is not related to gingivitis, halitosis and periodontitis.

## Conclusion

More males reported to the Dental Unit of the University Hospital than females and this is probably due to the University population profile or the fact that men are more predisposed to dental problems due to poor attitudes to oral health and its practice.

Although the majority (79.1 %) of patients have no significant past medical history, about 16 % have either hypertension, diabetes or both and these chronic conditions are significantly associated with related to apical periodontitis, enamel caries and the severity of presenting complaint.

The factors of age, duration of symptoms and the presence of multiple chronic conditions is related to the diagnoses arrived at during the dental visit.

More people presenting at the University Hospital come with acute dental conditions for emergency treatment. Most of the patients present after about 1 month of experiencing symptoms. Also fewer people are attending the clinic for preventive oral health care.

This raises an important point for policy formulators to firstly step up oral health education and prevention services as well as prepare the dental clinic adequately to deal with the expected numbers of acute cases that will present.
